# Essential Roles of Epithelial Bone Morphogenetic Protein Signaling During Prostatic Development

**DOI:** 10.1210/en.2013-2054

**Published:** 2014-04-14

**Authors:** Akiko Omori, Shinichi Miyagawa, Yukiko Ogino, Masayo Harada, Kenichiro Ishii, Yoshiki Sugimura, Hajime Ogino, Naomi Nakagata, Gen Yamada

**Affiliations:** Department of Developmental Genetics (A.O., G.Y.), Institute of Advanced Medicine, Wakayama Medical University, Wakayama, 641–8509, Japan; Okazaki Institute for Integrative Bioscience (S.M., Y.O.), National Institute for Basic Biology, National Institutes of Natural Science, Okazaki, 444–8787, Japan; Department of Clinical Anatomy (M.H.), Graduate School of Medical and Dental Sciences, Tokyo Medical and Dental University, Tokyo, 113–8591, Japan; Department of Oncologic Pathology (K.I.), and Nephro-Urologic Surgery and Andrology (Y.S.), Mie University Graduate School of Medicine, Tsu, Mie, 514–8507, Japan; Department of Animal Bioscience (H.O.), Nagahama Institute of Bio-Science and Technology, Nagahama, Shiga, 526–0829, Japan; and Division of Reproductive Engineering (N.N.), Center for Animal Resources and Development (CARD), Kumamoto University, Kumamoto 860–0811, Japan

## Abstract

Prostate is a male sex-accessory organ. The prostatic epithelia consist primarily of basal and luminal cells that differentiate from embryonic urogenital sinus epithelia. Prostate tumors are believed to originate in the basal and luminal cells. However, factors that promote normal epithelial differentiation have not been well elucidated, particularly for bone morphogenetic protein (Bmp) signaling. This study shows that Bmp signaling prominently increases during prostatic differentiation in the luminal epithelia, which is monitored by the expression of phosphorylated Smad1/5/8. To elucidate the mechanism of epithelial differentiation and the function of Bmp signaling during prostatic development, conditional male mutant mouse analysis for the epithelial-specific Bmp receptor 1a (*Bmpr1a*) was performed. We demonstrate that Bmp signaling is indispensable for luminal cell maturation, which regulates basal cell proliferation. Expression of the prostatic epithelial regulatory gene *Nkx3.1* was significantly reduced in the *Bmpr1a* mutants. These results indicate that Bmp signaling is a key factor for prostatic epithelial differentiation, possibly by controlling the prostatic regulatory gene *Nkx3.1*.

The prostate is a male sex-accessory organ for secreting seminal fluids. The prostate retains the potential for cell proliferation, differentiation, and morphogenesis, and it is frequently prone to benign and malignant tumor formation (1, 2). Although prostate cancer is the most frequently diagnosed cancer in men, the mechanisms of normal prostatic epithelial development and cell proliferation have not been well elucidated.

The initial outgrowth of the prostatic bud develops from the urogenital sinus in mice from embryonic day (E) 16.5 (E16.5) to E17.5 (3). After the prostatic budding, branching morphogenesis follows at approximately postnatal day (P) 14 (P14) (4). Concurrent with branching morphogenesis, epithelial cell differentiation is prominent at P14–P21 (1, 4). Prostatic organogenesis and cell differentiation become mature around puberty at P21–P28, when circulating androgen levels start to rise sharply (5, 6). Development of the prostatic gland depends on coordinated interactions between epithelia and stroma. Several growth factors such as Wnt, Sonic hedgehog (Shh), and bone morphogenetic protein (Bmp) are expressed during prostatic development (7–11). Most functional analyses of these growth factors have been performed on early prostatic morphogenesis. However, the functions of these growth factors (particularly Bmp signaling) for prostatic epithelial differentiation remain unclear.

Bmp signaling regulates diverse physiological events such as cell proliferation, differentiation, and apoptosis during organogenesis and pathogenesis (12, 13). Bmp ligands are bound to a membrane-bound heterodimer receptor complex, which contains a Bmp type-2 receptor (Bmpr2) and one of two Bmp type-1 receptors (Bmpr1a [Alk-3] or Bmpr1b [Alk-6]). Ligand binding to the receptor leads to phosphorylation of Sma- and Mad-related protein (Smad)1, Smad5, and Smad8. Phosphorylated Smads (pSmads) associate with regulatory Smad4 and translocate into the nucleus activating its target genes (14).

Bmps have been implicated in the regulation of prostatic branching morphogenesis and tumor growth (15–17). Bmp4 is expressed in the urogenital sinus mesenchyme of the bladder neck before the prostatic budding (18). Bmp7 is expressed in the urogenital sinus mesenchyme at E15, and its expression shifts to the prostatic epithelia in parallel with the progression of prostatic maturation (16). The importance of Bmp signaling has been shown by *Bmp4* haploinsufficient mutant studies and corresponding *Bmp7*-knockout mice (16, 18). Both mouse models exhibit increased prostatic branching. Bmpr1a mediates various functions during development of urogenital organs derived from urogenital sinus (19, 20). However, no mutant mouse models of the Bmp receptors have been investigated for prostatic development. Hence, the activities of Bmp signaling and its prostatic-specific functions have not been determined.

Prostatic epithelia contain primarily luminal and basal cells. Both cell types are considered to originate from immature urogenital sinus epithelium. Urogenital sinus epithelium is positive for keratin (K) K5/K14/K19, K8/K18, and p63 (21, 22). The epithelial cells differentiate into the columnar luminal epithelial cells, which exclusively express K8/K18 and exhibit androgen-dependent growth and secretory functions (21–23). By contrast, the basal cells express p63, K5/K14, and K19, and they have been suggested to include the multipotent progenitor cells (21, 23, 24). The coordinated differentiation of basal and luminal cells is essential for maintenance of homeostasis in the prostatic epithelium. Hence, dysregulated differentiation may be associated with abnormal epithelial status, such as epithelial hyperplasia or tumor formation (2, 25–27).

The mechanisms of prostatic epithelial differentiation have just begun to be investigated, and several genes are suggested as potential regulators (28–30). Among these potential regulators, the *Nkx3.1* homeobox gene is particularly intriguing because *Nkx3.1*-null mice display epithelial hyperplasia and impairment of secretory functions (31–33). The expression of *Nkx3.1* starts just before prostatic budding and is maintained into adulthood (31, 33, 34). It has been reported that androgen signaling regulates the expression of *Nkx3.1* (33, 35, 36). However, other factors may possess the potential to regulate *Nkx3.1* expression, including growth factor signaling.

In this study, we analyzed epithelium-specific conditional *Bmpr1a* mutants and showed that Bmp signaling was indispensable for luminal cell differentiation. The mutants exhibited abortive epithelial folding with augmented basal cell proliferation and defects in secretory protein production. Intriguingly, the expression of *Nkx3.1* was significantly reduced in the mutants. It is suggested that epithelial Bmp signaling plays a pivotal role in prostatic epithelial cell differentiation, possibly through controlling genes including *Nkx3.1*.

## Materials and Methods

### Mutant mice and the tamoxifen-inducible conditional-mutation strategy

The tamoxifen-inducible, knock-in mouse line *Shhtm2 (cre/ERT2)Cjt* (designated as *ShhCreERT2*) (37) was used to induce the conditional mutation of *Bmpr1atm2.1Bhr* (designated as *Bmpr1a-flox*) (38). To analyze the cell lineage of *Cre*-expressing cells, the reporter strain *Gt(ROSA)26Sortm1*(*EYFP*)Cos (designated as *R26R-YFP*) (39, 40) was utilized. Mouse strains C57BL/6JJcl and ICR were purchased from CREA and SLC, respectively. To inactivate *Bmpr1a* in the urogenital sinus epithelial cell lineage, we intercrossed *ShhCreERT2*/+*;Bmpr1a*+/− mice with *Bmpr1a-flox/flox* mice. Conditional-knockout (CKO) male mice (*ShhCreERT2*/+*;Bmpr1a-flox*/−, hereafter designated as *Bmpr1a*-CKO) were born with a normal Mendelian frequency. The epithelial morphology of *Bmpr1a-flox*/− and *ShhCreERT2*/+*;Bmpr1a-flox*/+ was normal and these lines were used as control specimens. The time of noon on the day when a vaginal plug was detected was designated as E0.5. The tamoxifen-inducible Cre-recombinase system removed the floxed sequence of the target gene (41). Tamoxifen (Sigma) was dissolved in sesame oil (Kanto Chemical) to a final concentration of 20 mg/mL. Four milligrams of tamoxifen per 40 g body weight was administrated (ip) to the pregnant mice at E9.5 (19). Tamoxifen-treated dams often experience dystocia and their pups died (data not shown) (42). Thus, tamoxifen-treated newborn pups were collected by cesarean-operation and were foster nursed (untreated) with ICR females. The number of 5–7 offspring per foster mother was adjusted. Offspring for each experiment were collected from at least 3 pregnant females. Prostate specimens of anterior prostate (AP) for histologic analysis were collected from control and Bmpr1a CKO mice, respectively, at 1 week of age (n = 3), 2 weeks of age (n = 4), 4 weeks of age (n = 6), 7 weeks of age (n = 3), and more than 6 months of age (n = 8). All experimental procedures were approved by the DNA Recombination Experiment Committee and Animal Care and the Use Committee of Wakayama Medical University, Wakayama, Japan.

### Prostate tissue isolation

Tissues were harvested as described previously and fixed in 4% paraformaldehyde at 4°C (19). The tissue specimens were then dehydrated, embedded in paraffin, and sectioned with thickness of 6 μm. For cryosections, harvested tissues were directly embedded into Tissue-Tec OCT compound (Lab-Tek Products Division) and sectioned with a thickness of 10 um.

### Histologic and immunofluorescence analysis of prostate tissue

For histologic analysis, the prostate tissue sections were stained with hematoxylin and eosin. For immunohistochemical analysis, antigen retrieval was performed by incubating the prostate tissue sections mounted on slides in 0.1 mM citrate buffer (pH 6.0) in an autoclave (121°C) for 1 minute and inactivating endogenous peroxidase activity in methanol containing 3% H_2_O_2_. For cryosection samples, they were fixed with 4% paraformaldehyde on ice 10 minutes and then used without any antigen retrieval. The primary antibodies, dilutions, and sources were as follows: anti-p63 (1:100, Santa Cruz Biotechnology); anti-Bmpr1a (1:100, Santa Cruz; and 1:100, Orbingen); anti-Nkx3.1 (1:100, Santa Cruz; 1:1000, kindly provided by Dr. Cory Abate-Shen, Columbia University Medical Center, New York, NY [43]); anti-phosphorylated-Smad1/5/8 (1:100, Cell Signaling Technology); anti-GFP (1:100, Abcam; and 1:100, Roche,); anti-CK19 (1:100, Abcam); anti-K8 (1:1000, Covance; and 1:50, Progen); anti-K14 (1:1000, Covance); and anti-Ki67 (1:100, NovoCastra). Prostate tissue sections were incubated with primary antibodies diluted blocking buffer overnight at 4°C. Final visualization was performed using the streptavidin-biotin system with diaminobenzidine as the final chromogen. The tissue sections were counterstained with hematoxylin for 20 seconds. Antibody detection and visualization was carried out using immunofluorescence with Alexa fluor 488 anti-mouse-IgG (Life Technologies) and Alexa fluor 546 anti-rabbit-IgG (Life Technologies). Tissue sections were counterstained with Hoechst 33342 (Sigma).

### Cell line analysis

The human prostate cancer cell-line PC3 was obtained from the RIKEN BioResource Center, Tsukuba, Japan. The PC3 cell line was maintained in RPMI-1640 medium (Wako) supplemented with 10% fetal bovine serum (Thermo Trace).

### Quantitative real-time PCR

Changes in gene expression were quantified using the 7500 real-time PCR system (Life Technologies) according to the manufacturer's instructions. Total RNA (1 μg) was isolated with ISOGEN (Nippongene) from the whole AP at P28, and from the bladder-neck region at P1. Synthesis of cDNA was carried out with the SuperScript III (Life Technologies) or Primescript RT (Takara) reagent kit, and the resulting cDNA was mixed with SYBR premix Ex Taq (Takara). Three pools of samples per group at P1 were tested in triplicate. Six samples were tested at P28. Statistical analysis was performed using Student's *t* test or Welch's *t* test followed by an *F* test (*P* < .05 was considered significant). The error bars given for the data represent SE. The relative RNA equivalents for each sample were determined by comparison with the levels of the normalized standard Mouse ribosomal protein L8 (*mrpL8*), and then calculated as the fold-change compared with the normalized standard. The following primers were used. *Nkx3.1*: forward, 5′-GTCAGCCCAAGCTAACCAGCA-3′; reverse, 5′-ACACACACACATCTGTGGATGGAA-3′; *Probasin*: forward, 5′-ACACTGCATGTGCTAGGCGT-3′; reverse, 5′-TCCCACACAAAATGTGACGG-3′ (44); *mrpL8*: forward, 5′-ACAGAGCCGTTGTTGGTGTTG-3′; reverse, 5′-CAGCAGTTCCTCTTTGCCTTGT-3′ (45).

### Plasmid DNA reporter constructs and luciferase assay

Genomic sequences of the 3′-region of the *Nkx3.1* locus, which includes the prostatic regulatory region (46), were obtained from NCBI (http://www.ncbi.nlm.nih.gov/nuccore/) and were submitted for analysis by rVISTA (http://genome.lbl.gov/vista) and MultiPipmaker (http://pipmaker.bx.psu.edu/pipmaker/). For promoter analysis, the genomic DNA fragments from C57BL were obtained by a standard PCR procedure and were inserted into the pGL4.24 vector (Promega Corp) using the Infusion system (Takara). Mouse Smad1 and Smad4 were amplified with RT-PCR and inserted into the pFLAG-cytomegalovirus vector (Sigma).

The expression and reporter plasmids were transfected into PC3 cells with Lipofectamine LTX plus (Life Technologies) according to the manufacturer's instruction. Twenty-four hours after transfection, luciferase activity was measured by chemiluminescence by employing the Dual-Luciferase Reporter Assay System (Promega Corp). The values were normalized against *Renilla* luciferase activity under the control of the cytomegalovirus promoter vector pGL4.74 (Promega). Further addition of Bmp7 (50 ng/mL) (R&D system) was treated after 16–20 hours after transfection of Smad1/4 expression vector, and samples were collected and their luciferase activities measured after 24 hours. More than 3 independent experiments were performed. Statistical analysis was performed using Student's *t* test or Welch's *t* test followed by an *F* test (*P* < .05 was considered as significant).

### Chromatin immunoprecipitation (Chip) assay

To isolate chromatin from prostate tissue cells, the ChIP assay kit (Upstate Biotechnology) with Dynabead Protein G (Life Technologies) was used. The bladder-neck region containing the prostate of ICR mice was dissected from the pups at postnatal day 2. pSmad1/5/8 (Cell Signaling Technology) and acetyl-histone H3 (Upstate) antibodies (2 μg) were used for immunoprecipitation. For mock control, rabbit Ig (Dako) was used. More than 3 independent experiments were performed. PCR was performed with the following primers:

*C1*: forward, 5′-CAACTCCTCTACCAGGATTAGACAGAG-3′; reverse, 5′-TGCATGCGCATTTTAGTGAAACTC-3′.

*C2*: forward, 5′-AGGTCCGAAGCTGAATTGC-3′; reverse, 5′-CCCACTGAAAACCAACCAAT-3′.

## Results

### Activation of Bmp signaling during prostatic epithelial differentiation

**Figure 1.**
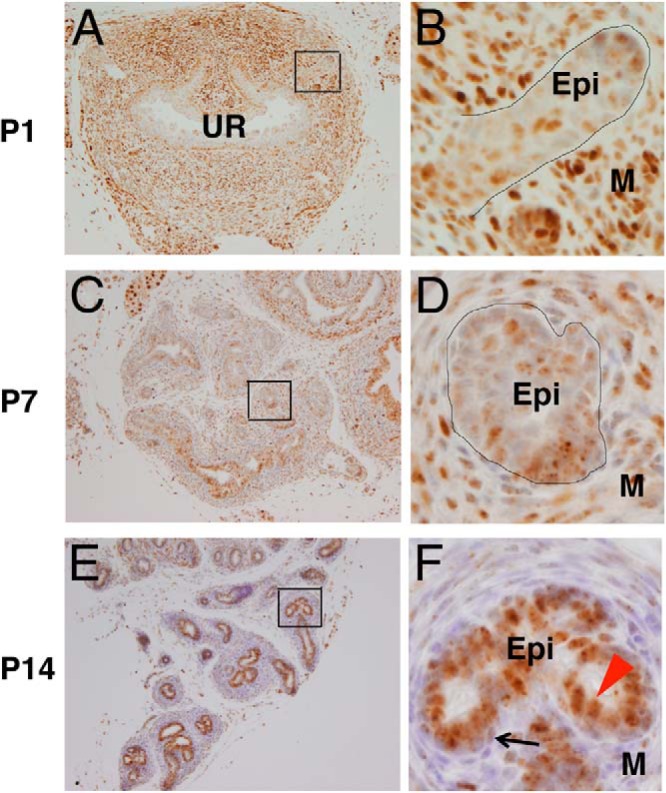
Bmp signaling was enhanced with the prostatic epithelial cell differentiation. A and B, Coronal sections of caudal body of ICR mice at P1; pSmad1/5/8 was weakly detected in the immature prostatic epithelia. C–F, Coronal sections of AP at P7 (C and D) and P14 (E and F). E and F, pSmad1/5/8 signal was more prominently observed in the differentiated luminal cells (F, red arrowhead) than that of the basal cells (F, black arrow) in the AP. UR, urethra. Epi, epithelia; M, mesenchyme. Scale bars, 100 μm.

To examine the role of Bmp signaling during prostatic development, we investigated the spatiotemporal activation of Bmp signaling by monitoring the expression of pSmad1/5/8 via immunohistochemical analysis (Figure 1). We detected pSmad1/5/8 expression in the prostatic bud at P1 (Figure 1, A and B), when immature stratified epithelium forms in prostate development (16). That signal was weakly detected in the prostatic epithelia at P7 (Figure 1, C and D). Such stage is before the starting of prostatic secretion (5). The expression of pSmad1/5/8 became prominent at P14 (Figure 1, E and F) as epithelial differentiation proceeded (5). At this stage, most pSmad1/5/8-positive cells were luminal cells (Figure 1F inset, red arrowhead), with some weakly positive basal epithelial cells (Figure 1F inset, black arrow). By contrast with such epithelial expression, the stromal expression of pSmad1/5/8 was comparatively low at this stage (Figure 1, E and F). This expression pattern suggests that epithelial Bmp signaling may function during prostatic development.

**Figure 2.**
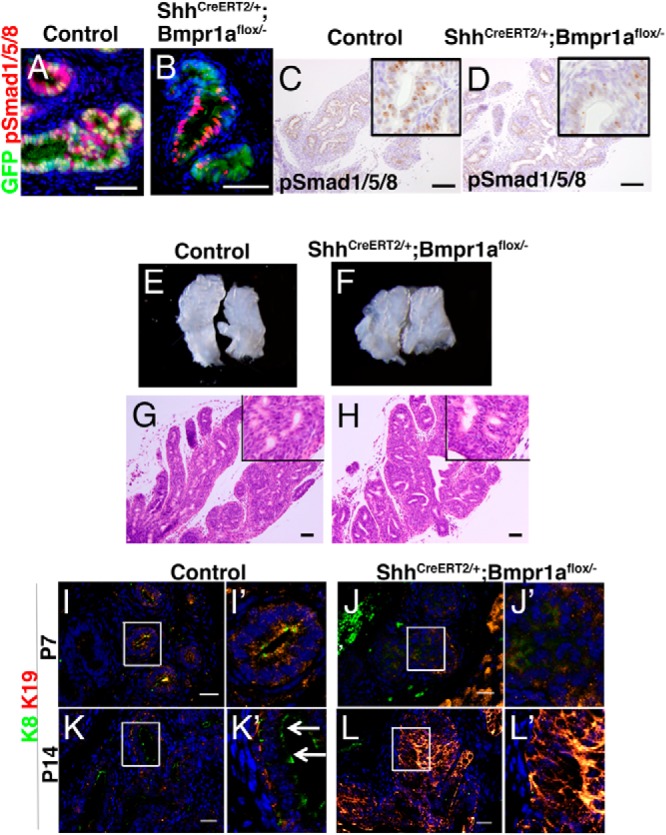
Defective prostatic epithelial differentiation was induced in the *Bmpr1a*-CKO mutants prostate. A, C, E, G, I, and K, Epithelial differentiation in the AP of control mice. B, D, F, H, J, and L, Epithelial differentiation in the AP of epithelial-specific *Bmpr1a*-CKO mutant mice. A and B, Costaining for green fluorescent protein (GFP) and pSmad1/5/8 in the AP at P14. Mosaic expression of GFP was detected only in the AP epithelia but not in the stroma of both control and *Bmpr1a*-CKO specimens (green). A and C, pSmad1/5/8 was localized primarily in the prostatic epithelia. B and D, Decreased pSmad1/5/8 expression was detected in the *Bmpr1a-C*KO specimens. E, Control AP ducts at P14. F, *Bmpr1a*-CKO mutant AP at P14. G and H, Histologic section of the control (G) and *Bmpr1a*-CKO mutant prostate (H). I–L, Costaining for K19 and K8 at P7 and P14. K, K19 was localized in basal epithelia and K8 was localized in the luminal epithelia of the control mouse prostate at P14 (K′, white arrows). K8 and K19 were aberrantly coexpressed at P14 in the *Bmpr1a*-CKO specimens (L). A–D and G and H, scale bars, 50 μm. I–L, scale bars, 20 μm.

We generated an epithelial-specific, conditional mutant of *Bmpr1a* to analyze the potential role of Bmp signaling during development of the prostatic epithelium. To introduce the mutation for *Bmpr1a* specifically in prostatic epithelial cells, *Bmpr1a*-floxed mice were intercrossed with *ShhCreERT2* driver mice, and tamoxifen was administrated at E9.5. The Cre driver mice strain introduces the recombination in the endodermal urogenital sinus epithelium (19), which is the origin of prostatic epithelial (both basal and luminal) cells (1) but not in the stromal cells. The recombination was confirmed using *R26RYFP* indicator allele and monitoring the expression of yellow fluorescent protein in the AP at P14 (Figure 2, A and B). Although some epithelial cells did not show the recombination due to the mosaic Cre expression (Figure 2, A and B), The number of pSmad1/5/8-positive cells was significantly reduced among the Cre-expressing cells of *Bmpr1a*-CKO mice compared with that of control mice (Figure 2, A–D and Supplemental Figure 1, A–F). The reduced number of pSmad1/5/8-positive cells was counted (Supplemental Figure 1G). Thus, we judged that this system was adequate to analyze the specific role of Bmp signaling in prostatic epithelia.

### Defective prostatic epithelial cell differentiation in *Bmpr1a*-CKO mutant mice

**Figure 3.**
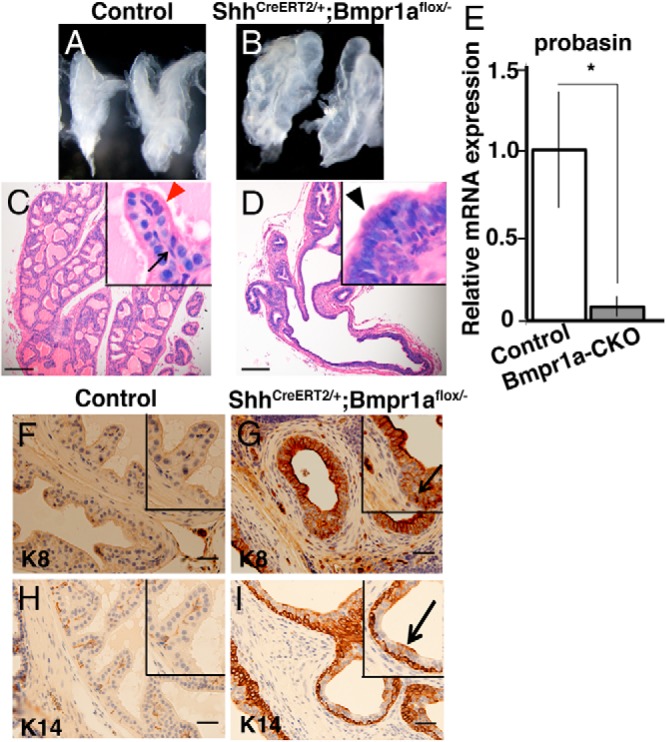
Abnormal stratification and decreased mRNA level of secretory protein in the *Bmpr1a*-CKO mutant prostate. A, Control AP ducts at P28. B, *Bmpr1a*-CKO mutant AP at P28. C, Histologic section of the control prostate. Inset, Luminal cells (red arrowhead) and basal cells (black arrow) were observed. D, Histology of the *Bmpr1a*-CKO mutant prostate. Inset, Abnormally stratified epithelia were observed (black arrowhead). Scale bars, 100 μm. E, Probasin mRNA expression was decreased in the mutant AP at P28. The relative RNA equivalents for each sample were normalized by the RNA levels for ribosomal protein L8. Error bars represent the mean ± SE of 7 tissue samples. Statistical significance was indicated by an asterisk. F and G, Expression of K8 in control (F) and mutant (G) prostate. K8-positive basal side epithelia were indicated (G, black arrow). H and I, Expression of K14 in control (H) and mutant (I) prostate. K14-positive luminal side epithelia were indicated (I, black arrow). Increased levels of K8 (G) and K14 (I) were detected in the *Bmpr1a*-CKO mutants at P49. Scale bars, 20 μm.

The AP of control and *Bmpr1a*-CKO mutant mice developed normally with respect to gross morphology and hematoxylin and eosin sections at stage P14, when prominent epithelial differentiation was observed (Figure 2, E–H). However, defective epithelial differentiation was detected in the mutant based on the expression of keratin markers. The expression of K19 was used as a marker of intermediate differentiated cells, and the expression of K8 was used as a marker of luminal epithelia (21, 22). In the control prostate, both K19 and K8 were coexpressed in the epithelia at P7 (Figure 2I). At P14, K19 and K8 were exclusively expressed in the basal and luminal epithelial cells, respectively, indicating normal cell differentiation in both cell types (Figure 2, K and K′ inset, white arrows). In mutants at P7, the expression of both K19 and K8 was lower than those of controls (Figure 2, J and J′). At P14, both K19 and K8 were strongly expressed and colocalized in the epithelia, which suggests a presence of intermediate cells (Figure 2, L and L′). Serum testosterone levels rise sharply during P21–P28 (at the stage of puberty), with a simultaneous increase of the weight of the prostate (1, 5, 6). Morphologic defects in the mutant prostates were evident at P28, and they displayed lower number of ducts with swelling structure compared with those of the controls (Figure 3B). Histologically, normal AP displayed distinct features such as epithelial-infolding structures that consisted of pseudostratified epithelium (Figure 3C). By contrast, these epithelial structures were reduced in the mutant AP (Figure 3D). The reduction in epithelial-infolding structures was also observed in the ventral and dorsal prostate of the mutant compared with that of the control (Supplemental Figure 2). The ductal epithelia in the mutant were multiply stratified with irregularly shaped cells (Figure 3D) compared with that of normal pseudostratified epithelia (Figure 3C). In the normal prostate, luminal cells exhibited a columnar lining in the luminal side (Figure 3C inset, red arrowhead), whereas basal epithelial cells were flattened forming a discontinuous layer of cells (Figure 3C inset, black arrow). These distinctive features of basal and luminal epithelial cells were not observed in the mutants (Figure 3D inset, black arrowhead). Furthermore, the lumen of normal prostatic glands displayed eosinophilic secretions, which were not observed in the lumen of the mutant prostate. The mRNA expression of the prostatic secretory protein, probasin, was significantly reduced in the mutant prostates compared with those of controls (Figure 3E).

K8 was weakly expressed in luminal epithelial cells of control mice at adult stage P49 (Figure 3F). In the *Bmpr1a*-CKO mutants, K8 expression was increased in the multiply stratified epithelia and was observed in the basal side epithelia (Figure 3G inset, black arrow). Expression of the K14 basal cell marker for prostatic epithelium was discontinuous in the region adjacent to the basal membrane in the AP of control mice (Figure 3H). By contrast, K14-positive cells were observed in the aberrant, multiple stratified epithelia of the mutant AP, and some epithelial cells in the luminal region were also positive for K14 (Figure 3I inset, black arrow). The coexpression of luminal and basal cell marker has been indicated as undifferentiated prostatic epithelia (21, 22). Thus, the aberrant expression pattern of luminal and basal cell markers may indicate the presence of defective epithelial differentiation in the mutant AP (Figure 3, F–I). This defective basal-luminal differentiation in the mutant tissue was also detected at P14 (Figure 2L) and was observed with the aberrantly stratified epithelia at P28 (Supplemental Figure 1, H–K). It might cause the decreased prostatic secretory functions indicated by the reduced mRNA expression of probasin (Figure 3E).

### *Bmpr1a* conditional mutation leads to prostatic epithelial hyperplasia

**Figure 4.**
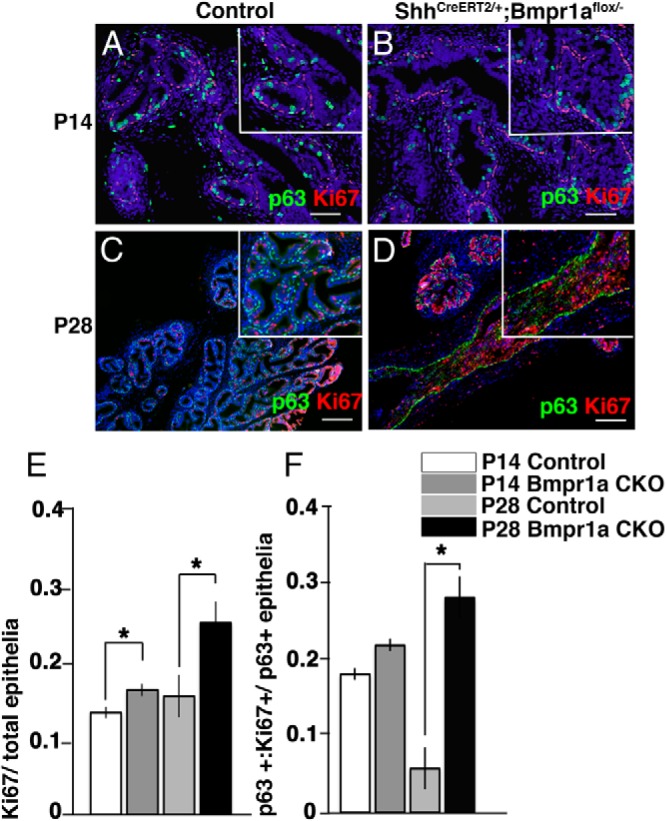
Basal cell proliferation increases in the *Bmpr1a*-CKO mutants. A–D, Costaining for Ki67 and p63 at P14 (A and B) and P28 (C and D) was performed. E, Ki67-positive epithelia were quantified at P14 and P28. F, Cells positive for both Ki67 and p63 were quantified at P14 and P28. Scale bars, 50 μm. Significantly increased ratios of proliferative epithelia (E) and proliferative basal cells at P28 (F) were detected. Data are presented as means of 3 values ± SE. Statistical significance was calculated using Student's *t* test followed by the *F* test (*, *P* < .05).

Cell proliferation in the *Bmpr1a*-CKO mutant prostate was analyzed by immunohistochemical analysis using antibody against Ki67, which is a marker for cell proliferation. Basal cells are considered to contain multipotent progenitors for postnatal prostatic development (23). Therefore, we examined Bmp signaling during basal cell proliferation based on the expression of both Ki67 and p63 (Figure 4, A–D). The total number of Ki67-positive epithelial cells was slightly increased in the mutant prostate compared with that of the control prostate at P14 (Figure 4E). At P28, the total number of Ki67-positive cells was augmented in the mutants (Figure 4E). Among the p63-positive basal epithelial cells, no significant differences in cell proliferation were observed between the control and *Bmpr1a*-CKO tissues at P14 (Figure 4F). The p63 epithelial cell proliferation in controls appeared to be decreased at P28, whereas *Bmpr1a*-CKO showed a higher level of proliferation. It was sustained at a high level as shown in P14 specimens (Figure 4F). Prominent prostatic tissue growth and expansion occur at approximately P28 and are correlated with the increase in androgen levels (6). The significant increase in cell proliferation of the basal cells might arise from the abnormally differentiated cells detected in the mutants. These data suggest that Bmp signaling regulates epithelial cell proliferation via Bmpr1a, demonstrating that mutation of *Bmpr1a* leads to prostatic epithelial hyperplasia.

### Expression of prostatic *Nkx3.1*, the key epithelial regulatory gene, was significantly reduced in the *Bmpr1a* mutant prostate

**Figure 5.**
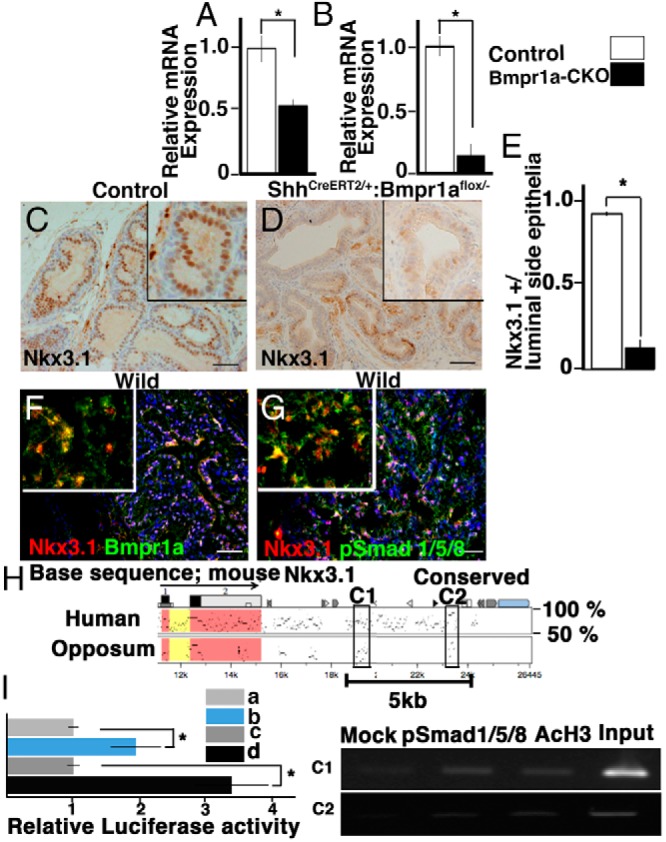
Decreased expression of *Nkx3.1* in the *Bmpr1a*-CKO mutants. A and B, *Nkx3.1* expression decreased in mutant prostate at P1 (A) and in the mutant AP at P28 (B). The relative mRNA equivalents for each sample were normalized by the RNA levels for ribosomal protein L8. Bars represent the mean ± SE of triplicate assays of RNA from pooled tissues (A) and 6 tissue samples (B). Statistical significance was indicated by asterisks (*, *P* < .05). C, Nkx3.1 was detected in the luminal epithelia of the control AP at P28. D, Significantly reduced levels of Nkx3.1 protein were detected in the mutant AP. E, The ratios of Nkx3.1-positive cells were shown in the graph. F, Colocalization of Nkx3.1 and Bmpr1a in the AP luminal epithelia of adult mice. G, Colocalization of pSmad1/5/8 and Nkx3.1 in the AP luminal epithelia of adult mice. F and G, Cryosections were used. Scale bars, 20 μm. H, Genomic sequences of the mouse *Nkx3.1* aligned with its orthologous loci in human and opossum. The sequence alignment was performed using MultiPipMaker. A noncoding region conserved from human to opossum was indicated with black boxes in the C1 and C2 regions. The black arrow indicated exons of mouse *Nkx3.1*. Coding and untranslated sequences were shaded with red and yellow, respectively. A 5-kb (5399 base) region in the 3′-genomic region of *Nkx3.1* contained a candidate enhancer region for the mouse prostate. The scale at the bottom of the alignment indicated relative positions in the mouse *Nkx3.1* locus. I, The candidate 5-kb prostatic regulatory enhancer activated expression of a luciferase reporter in response to Smad1/4 expression (by 6 independent assays). It also responded to the addition of Bmp7 (by 3 independent assays) (means ± SE) (*, *P* < .05). a, Control. b, Transfected with Smad1/4 gene. c, Control. d, Transfected with Smad1/4 gene + addition of Bmp7. J, ChIP/PCR assay on bladder neck of ICR mice including prostate region at P2 showed pSmad1/5/8 binding to regions of C1 and C2 in the 3′-region of mouse *Nkx3.1*. Both regions were enriched in chromatin immunoprecipitated with antiacetylated histone H3 as a positive control.

To further investigate the role of Bmp signaling in prostatic epithelial differentiation, expression of several epithelial cell-regulatory genes was quantified with real-time RT-PCR in control and *Bmpr1a*-CKO mutant prostatic tissues. The genes included *Foxa1* (30), *Nkx3.1* (31), and *Notch1* (29). Among such genes, the expression of *Nkx3.1* was reduced in the mutants at P1 and P28 compared with the levels in controls (Figure 5, A and B). We then assessed the protein levels of Nkx3.1 and Bmpr1a in the prostate. The levels of Nkx3.1 in the nuclei of luminal epithelial cells were decreased in the *Bmpr1a*-CKO mutant mice compared with those in the controls at P28 (Figure 5, C–E). Bmpr1a and Nkx3.1 proteins were coexpressed in the luminal epithelia of the normal adult prostate (Figure 5F). Consistently, pSmad1/5/8 was coexpressed with Nkx3.1 in the luminal epithelia of normal adult prostate (Figure 5G). These results suggest that the decreased expression of *Nkx3.1* in the *Bmpr1a*-CKO mutants is possibly due to the reduction of Bmp signaling.

### Bmp signaling in epithelia may regulate *Nkx3.1* expression in the prostate

To examine the possible Bmp-dependent regulation of *Nkx3.1* expression, reporter assays and chromatin immunoprecipitation were performed. The genomic *Nkx3.1* locus contains a candidate enhancer region for the mouse prostate, which is located at −7 kb from the 3′-coding region of *Nkx3.1* (46). Using the rVISTA genome browser and MultiPipmaker (47), we found that the 3′-region of *Nkx3.1* was conserved among mammals but not in other vertebrates (data not shown). The conserved region was illustrated by a comparison of the human, mouse, and opossum loci (Figure 5H), which contained several Smad-binding elements. The opossum genome is placed at an evolutionary midpoint relative to eutherian mammals and nonmammalian vertebrates. Therefore, this analysis facilitates the evaluation of regulatory mechanisms that may be shared between mammals (48, 49). We focused on the element that was highly conserved among the mouse, human, and opossum *Nkx3.1* gene locus. It was observed that the element responded to the expression of Smad1/Smad4 in the PC3 cell line (Figure 5I, a and b). The response was more enhanced by the addition of Bmp7 ligand, which is known to be expressed during prostatic development and homeostasis (15, 16) (Figure 5I, c and d). A chromatin immunoprecipitation assay was performed, followed by PCR using primer regions C1 and C2 (Figure 5H) for the 3′-genomic fragment of *Nkx3.1* within the Smad-binding element. Smad-specific enrichment was observed in the extracts from the bladder-neck tissues of ICR mice at P2 (Figure 5J). Taken together, these results suggest that Bmp signaling may regulate *Nkx3.1* expression via Smads.

### Conditional mutation of *Bmpr1a* in epithelia leads to stromal hyperplasia and inflammation

**Figure 6.**
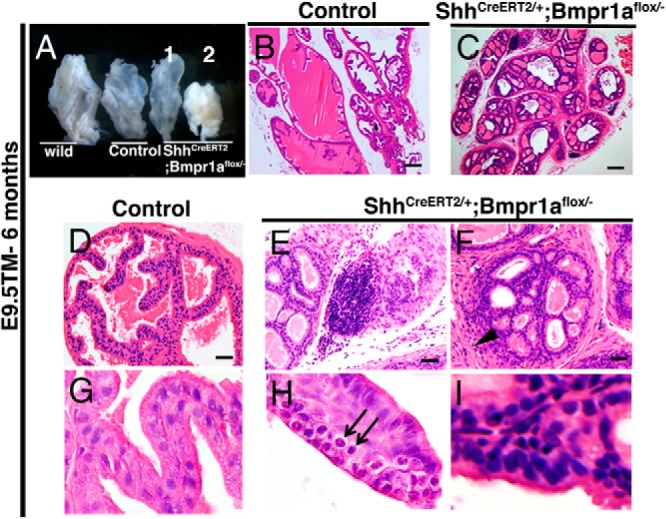
Stromal hyperplasia and inflammation with enlarged epithelial nuclear structures were observed in the *Bmpr1a*-CKO mutants. A, Morphology of AP in wild-type, *ShhCreERT2*/+;*Bmpr1a-flox*/+ (Control), and *ShhCreERT2*/+; *Bmpr1a-flox/*− mice at 6 months of age. B, D, and G, Histology of control AP. C, E, F, H, and I, The sections of the epithelial-specific *Bmpr1a*-CKO mutant AP. B–I, Sections stained with hematoxylin and eosin showed inflammatory cells in the stroma (E), stromal hyperplasia (F, black arrow head), inflammatory cells (H, black arrows), and enlarged nuclear structures (I) in the AP of *Bmpr1a*-CKO mice. A and B, Scale bars, 200 μm. D–F, Scale bars, 20 μm.

Mutations in *Bmpr1a* in the human and mouse are responsible for organ pathogenesis with abnormal cell proliferation, such as juvenile polyposis syndrome and skin tumorigenesis (50–52). However, the role of *Bmpr1a* in prostatic cancer has not been elucidated. Null mutants of *Nkx3.1* display prostatic intraepithelial neoplasia (PIN) associated with tumorigenesis at 6 months after birth (53). Thus, we investigated whether the loss of function of *Bmpr1a* with the decreased level of *Nkx3.1* induces prostatic carcinogenesis. We dissected the mutant prostate samples from *Bmpr1a*-CKO mice at 6 months of age (Figure 6A). At this age, the *Bmpr1a*-CKO prostates displayed cribriform ducts with intraepithelial inflammatory cells (Figure 6, C and H, black arrows), stromal hyperplasia (Figure 6F, black arrowhead), and the enlarged nuclei of the epithelia (Figure 6I) compared with those of the control specimens (Figure 6, B, D, and G). However, nuclear atypia of epithelial cells, which are a hallmark of PIN (53), were not detected in the prostates of the *Bmpr1a*-CKO mutants (Figure 6, C, E, and F, and H and I).

## Discussion

Bone morphogenetic protein signaling mediates multiple physiological functions during organogenesis and pathogenesis (13, 15). Previous studies revealed an inhibitory role for Bmp signaling on branching morphogenesis of the prostate. However, the functions of Bmp signaling during prostatic epithelial cell differentiation are unknown. The current study detected the expression of phosphorylated Smad1/5/8 (Bmp signaling mediators) in prostatic epithelial cells. An epithelial cell-specific conditional mutant for *Bmpr1a* exhibited defective prostatic epithelial differentiation. One possible mechanism of Bmp signaling during epithelial differentiation may be mediated by *Nkx3.1*, which is a homeobox gene that regulates prostatic epithelial cell differentiation and development, because a significant reduction of *Nkx3.1* expression was detected in the *Bmpr1a*-CKO mutants. These data indicate that Bmp signaling is indispensable for prostatic epithelial cell maturation during prostatic development.

It has been suggested that cells coexpressing the K5/K14/K8/K18/K19 and the p63 are epithelial progenitors in the urogenital sinus epithelium and adult epithelia (21, 54). These progenitor cells differentiate into K5/K14/p63/K19-positive basal cells and K8/K18-positive luminal cells (21, 23). During this process, differentiating luminal cells develop into K8/K19-positive intermediate cells (21, 22). Basal cells are considered to possess multipotent characters and they can differentiate into the luminal cells during postnatal prostatic development (23). According to this model, coexpression of basal and luminal markers indicates that the cell is in an undifferentiated state (21, 22). However, the effector signals that promote prostatic epithelial differentiation have not been well identified.

The current study monitored Bmp signaling by the expression of pSmad1/5/8 using immunohistochemistry. Prominent expression of pSmad1/5/8 was detected in the differentiated luminal epithelia, whereas its expression in basal cells was comparatively low. In addition, defective luminal cell differentiation and protein secretion were observed in the prostatic epithelia of *Bmpr1a*-CKO mutants. These data may indicate Bmp signaling is required for luminal cell differentiation from the prostatic progenitor cells. The aberrant coexpression of intermediate markers, K8 and K19, in the *Bmpr1a*-CKO mutants might indicate that Bmp signaling is required for epithelial differentiation of intermediate cells to luminal cells. The function of Bmp signaling mediated via Bmpr1a for the epithelial differentiation has been reported in various organs such as intestine, lung, and hair follicle (55–57). The current results suggest Bmp signaling also plays a critical role for epithelial differentiation during prostatic development.

Defective epithelial cell differentiation was observed at P14, when the androgen level is low compared with that at puberty (1, 6). An aberrant increase in basal cell proliferation was observed at P28, when prostatic tissue displays significant increases in growth in response to androgen levels (1, 6). In contrast, decreased basal cell proliferation was observed in the control at P28 compared with specimens at P14, indicating that the mature basal cells are not highly proliferative. In the *Bmpr1a*-CKO mutants, the highly proliferative p63-positive cells might be derived from immature basal cells or the abnormally differentiated intermediate cells observed at P14. Alternatively, they might be derived from other abnormally differentiated cells, because the differentiation of basal cells to luminal cells also occurs at later stages than P14 (23). The correct regulation of cell differentiation-mediated Bmpr1a before the increase of androgen levels would be crucial for proper prostate maturation and for inhibiting augmented basal cell proliferation at puberty.

### Bmp signaling may regulate prostatic epithelial differentiation through Nkx3.1

*Nkx3.1* is a vertebrate homeobox gene that plays key roles in prostatic epithelial cell differentiation (31, 33, 34). Loss of *Nkx3.1* function in mice results in the reduced synthesis of secretory proteins and epithelial hyperplasia and dysplasia (31, 32, 58). Thus, *Nkx3.1* is essential for prostatic epithelial cell maturation. In the current study, loss of Bmp signaling in the prostatic epithelia of *Bmpr1a*-CKO mutants led to reduced expression of *Nkx3.1*. It has been known that the *Nkx3.1* expression levels are higher in the AP compared to other lobes (31). Accordingly, such high expression of *Nkx3.1* may induce the current mutant epithelial phenotypes primarily in the AP.

The testosterone level in the testis was not significantly different in the control and *Bmpr1a*-CKO mutants (Supplemental Figure 3). However, a reduced level of the epithelial androgen receptor (AR) was observed in the mutant prostate (Supplemental Figure 3). AR signaling has been suggested to regulate *Nkx3.1* expression (31, 33, 35, 36). Therefore, the decreased expression of *Nkx3.1* could be also due to down-regulation of AR in the *Bmpr1a*-CKO mutants. Although circulating androgen levels display 2 peaks during prostatic development (1, 6), *Nkx3.1* expression levels and patterns might be altered independently from the androgen levels during development (6, 10, 59). Other signals may be involved in regulating *Nkx3.1* gene expression. In the current study, regulatory element analyses using a human prostatic carcinoma cell line and ChIP analyses using mouse prostatic tissue suggest that Bmp signaling may directly regulate *Nkx3.1* expression. These results suggest that Bmp signaling could be one of the possible regulatory factors of *Nkx3.1* expression. Further work is required to elucidate the coordinated role of AR and Bmp signaling on *Nkx3.1* gene expression.

### *Bmpr1a*-CKO mice display stromal hyperplasia and inflammation without PIN phenotypes

Previous reports suggest a suppressive role of Bmp signaling for tumorigenesis in several organs. Inactivation of Bmpr1a in skin can induce the formation of tumors in hair follicles, and epithelial ablation of *Bmpr1a* correlates with the occurrence of juvenile polyposis with a risk for colon cancer (56, 57). Some in vitro studies indicate that Bmp7 and other Bmp ligands are potent inhibitors of tumor growth and metastasis in prostatic cancer cell lines (15, 60). However, there are no reports on Bmpr1a function in prostatic tumor by mouse genetic studies.

In addition to epithelial phenotypes, stromal hyperplasia was observed in the *Bmpr1a*-CKO mutant prostate. The epithelial-stromal interaction is important for development and homeostasis in prostatic tissue (2, 12). The defects of epithelial differentiation in the *Bmpr1a*-CKO mutants might induce secondary phenotypes such as stromal hyperplasia thorough aberrant paracrine signals. Our study suggests that epithelial Bmp signaling mediated via Bmpr1a is indispensable for normal stromal development.

The *Bmpr1a*-CKO mice display inflammation of prostatic epithelia with enlarged nuclei of the epithelial cells. However, we did not observe nuclear atypia of the epithelia, which is a hallmark of PIN (53). These data indicated that the *Bmpr1a*-CKO mutation did not lead to prostatic carcinoma, at least not at the age of 6 months. The *Nkx3.1-*null mutation can induce PIN at 6 months (31). Thus, ablation of *Bmpr1a* and the resulting reduction in *Nkx3.1* might not be sufficient to induce prostatic carcinoma. The coordinated roles of several factors including Nkx3.1, cMyc, β-catenin, phosphatase and tensin homolog, and Smad4 contribute to prostatic tumor progression (43, 61–64). Additional factors including Bmpr1a might be required for the induction of prostatic tumors. Future work should investigate the collaborative roles of Bmp signaling and additional prostatic regulatory genes.

Basal and luminal cells have been investigated as the origin for prostate tumor to examine which lineages acquire genetic alterations promoting such cancer initiation (24–26, 65, 66). Furthermore, an increase in the number of intermediate cells in adult prostates is often indicative of several prostatic pathologic states (67, 68). However, the factors that promote epithelial differentiation and maintain the differentiated state of each cell type have not been extensively characterized. The current results suggest that Bmp signaling regulates cellular differentiation during prostatic development. This work may offer insight into the cellular behaviors underlying some prostatic pathologic states.
